# Recent advancements in application of carbohydrate-derived carbon quantum dots in analytical chemistry: a comprehensive update

**DOI:** 10.1186/s40643-025-00953-x

**Published:** 2025-10-31

**Authors:** Elyor Berdimurodov, Natarajan Elangovan, Ashish Kumar, Abhinay Thakur, Fotima Sobirova, Khudaybergan Polvonov, Sevara Tojieva, Muzaffar Makhkamov, Ahmad Hosseini-Bandegharaei, Ibrohim Sapaev

**Affiliations:** 1 Faculty of Chemistry, National University of Uzbekistan, 100034 Tashkent, Uzbekistan; 2 School of Medicine, Central Asian University, 111221, Tashkent, Uzbekistan; 3 Applied Sciences, University of Tashkent for Applied Sciences, 100149, Str. Gavhar 1, Tashkent, Republic of Uzbekistan; 4 Centre for Global Health Research, Saveetha Medical College,, Saveetha Institute of Medical and Technical Sciences, Chennai, India; 5 Nalanda College of Engineering, Science Technology and Technical Education Department, Government of Bihar, Bihar Engineering University, Patna, 803108 India; 6 Division of Research and Development, Lovely Professional University, Phagwara, , Punjab 144411 India; 7 Department of Pharmacy and Chemistry, Alfraganus University, 100190, Tashkent, Uzbekistan; 8 Natural and Agricultural Sciences, Urgench State University Named After Abu Rayhan Biruni, 220100 Urgench City, Uzbekistan; 9 Faculty of Chemistry and Biology, Karshi State University, Karshi City, Uzbekistan; 10 Faculty of Chemistry, Semnan University, Semnan, Iran; 11 Centre of Research Impact and Outcome, Chitkara University, Rajpura, Punjab 140417 India; 12 Department of Sustainable Engineering, Saveetha School of Engineering, SIMATS, Chennai, Tamil Nadu 602105 India; 13 Physics and Chemistry, “Tashkent Institute of Irrigation and Agricultural Mechanization Engineers” National Research University, Tashkent, Uzbekistan

**Keywords:** Carbon quantum dots (CQDs), Carbohydrate precursors, Fluorescent biosensors, Environmental monitoring, Food safety detection

## Abstract

Carbohydrate-derived carbon quantum dots (CDCQDs) have evolved at a rapid rate as green, biocompatible nanomaterial, revolutionizing analytical chemistry with their unique optical and surface properties. Synthesized from different carbohydrate sources-ranging from mono-, di-, and polysaccharides to biomass wastes-CDCQDs offer tunability of fluorescence, low toxicity, and simple functionalization, enabling ultrasensitive detection in chemical, biomedical, environmental, and food safety applications. Recent developments (2021–2025) have reached detection sensitivities of as low as 0.077 µM for antibiotics, 7 nM for glucose, and single-cell sensitivity for pathogens with recovery rates routinely > 95%. Quantum yield (QY) up to 83% and superb photostability also allow them to be included in portable and point-of-care platforms. Notwithstanding such accomplishment, scalability, reproducibility, and integration into devices continue to be issues. Mitigation of these via green synthesis, surface engineering, and smart device coupling is crucial for commercial translation. CDCQDs are thus a synthesis of sustainability, sensitivity, and versatility and are poised to drive next-generation eco-friendly analytical systems for real-world diagnostics and monitoring.

**Graphical abstract:**

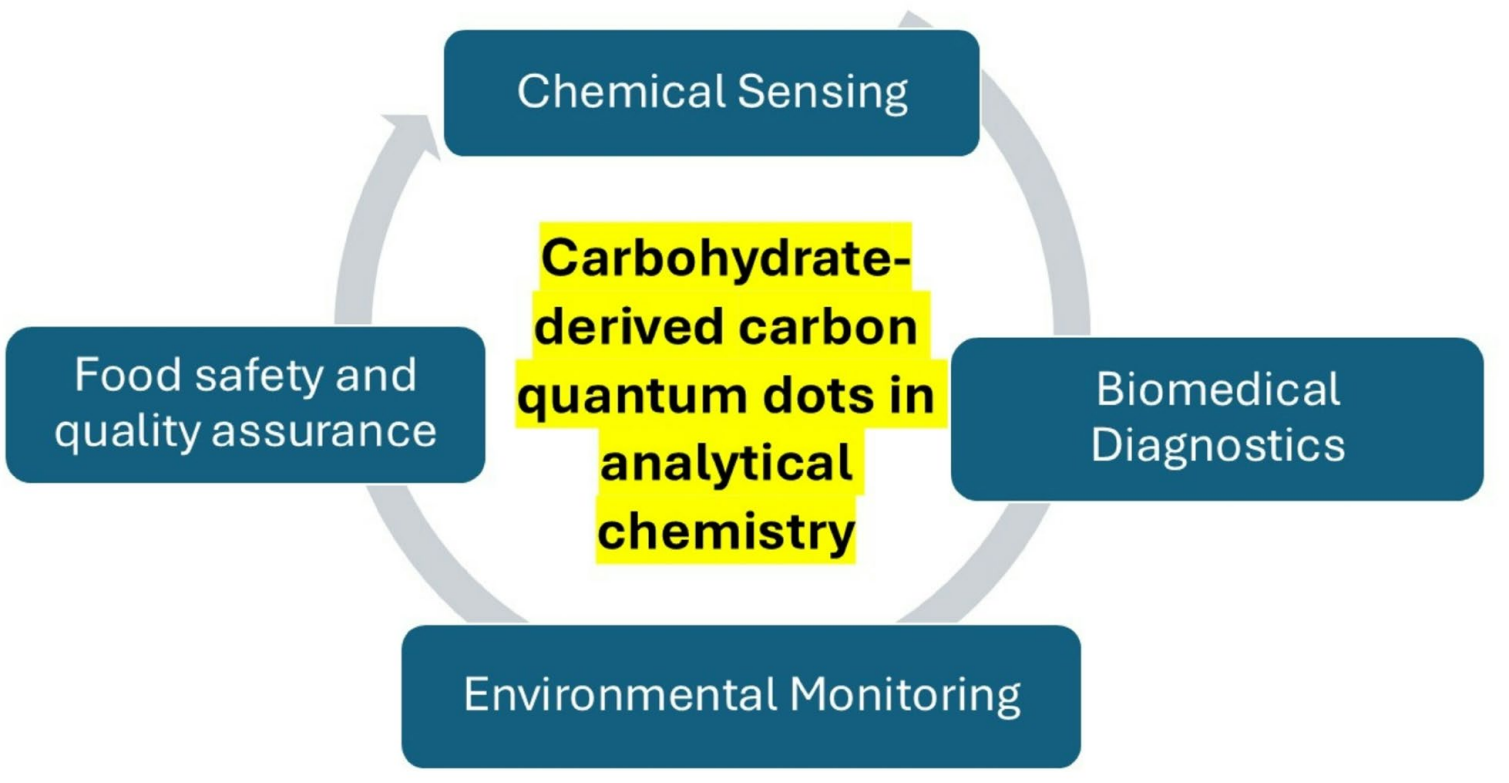

## Introduction

Carbon quantum dots (CQDs) are rapidly a highly acclaimed family of zero-dimensional carbon nanomaterials, well known for their ultrasmall size (typically < 10 nm), high water solubility, and excellent photostability. Their unique electronic structure by virtue of a mixture of sp^2^ and sp^3^ hybridized carbons bestows upon them tunable photoluminescence and allows for facile surface functionalization with a range of functional groups, e.g., –OH, –COOH, and –NH_2_, thus enhancing their chemical diversity and biocompatibility. These characteristics, in addition to their low toxicity and high QY, have brought CQDs to the forefront of analytical chemistry, where they drive innovation in chemical sensing, bioimaging, diagnostics, and environmental monitoring (Abbas et al. 2023; Abu et al. 2023; Arias Velasco et al. 2021).

Carbohydrate-source carbon quantum dots (CDCQDs) emerged to the fore at the frontiers of green nanotechnology by leveraging the intrinsic abundance, renewability, and structural variability of carbohydrate precursors from monosaccharides and disaccharides through complex polysaccharides to biomass residues (Baragau et al. 2021; Bijoy and Sangeetha 2024; Chellasamy et al. 2022). The high concentration of hydroxyl and carbonyl function content in the molecules not only facilitates efficient carbonization but also enables selective doping with heteroatoms and varied surface functionalization, thereby constructing the optical and electronic characteristics of the resulting CQDs. This environmentally benign synthesis technique not only minimizes environmental issues but also yields nanomaterials with high biocompatibility, tuneable emission, and steady analytical performance, making CDCQDs core materials for next-generation sensing and diagnostic devices.

The current review provides an extensive and up-to-date overview of carbohydrate-derived carbon quantum dots (CDCQDs) research from 2021 to 2025, with particular emphasis on their revolutionary role in analytical chemistry. It systematically describes the latest synthetic approaches, physicochemical characteristics, and advanced surface engineering techniques that significantly influence the analytical performance of CDCQDs. In highlighting these intrinsic features, the review describes how tailored synthesis and functionalization methods provide precise control over optical properties and biocompatibility, thereby increasing the utility of CDCQDs. The discussion spans a broad range of carbohydrate precursors, ranging from trivial monosaccharides to complex polysaccharides and biomass waste, to underscore the sustainable and green chemistry principles driving the field. This general overview serves as the basis for enjoying the varied uses of CDCQDs in different analytical contexts.

Building on this foundation, the review analyzes the practical applications of CDCQDs in chemical sensing, biomedical diagnosis, environmental monitoring, and food safety assurance. The focus is specifically directed towards fluorescence-based detection platforms, wherein CDCQDs are very selective, sensitive, and recyclable. The discussion covers an extensive array of target analytes-ranging from heavy metals, pesticides, biomolecules, antibiotics, dyes, and pathogens-spotting new trends in detection sensitivity, response time, and real-sample application. Besides, the review also addresses some of today's biggest issues such as reproducibility, scalability, and regulatory barriers while it examines likely prospects for eventual use of CDCQDs in portable intelligent sensor devices. With this inclusion of up-to-date literature, this not only signals the growing importance of carbohydrate nanomaterials but also enunciates a path towards commercialization and wider analytical exploitation.

## Synthesis and properties of CDCQDs

### Carbohydrate precursors

Carbohydrate-based carbon quantum dots (CDCQDs) constitute a paradigm case of the confluence of nanotechnology and green chemistry, which takes advantage of the structural versatility and abundance of carbohydrates to design functional nanomaterials of unparalleled analytical potential (Chen et al. 2021a; Dong et al. 2024; Fan et al. 2022). The selection of carbohydrate precursors exerts a significant influence on the optical and physicochemical properties of the resulting CQDs, enabling precise regulation of their size, surface functionality, and photoluminescent characteristics.

Monosaccharides such as glucose, fructose, and galactose are used because of their stable molecular structure and high density of hydroxyl groups, facilitating uniform carbonization and the generation of CQDs with normal size distribution and tunable fluorescence (Farid et al. 2024; Ganesan et al. 2022a). Disaccharides such as sucrose and lactose introduce additional complexity with glycosidic linkages, typically yielding CQDs with enhanced QY and surface functional groups due to intermediate reactions during synthesis. Polysaccharides like starch, cellulose, and chitosan not only provide a high-carbon skeleton but also functional groups of various types-such as amino groups in chitosan-thereby enabling heteroatom doping and advanced surface engineering, thus expanding the range of applications of CDCQDs in analysis (Hao et al. 2023; He et al. 2023; Jagannathan et al. 2021).

Notably, agricultural residues, fruit and vegetable peels, and other food wastes, which are used as feedstocks, are sustainable and inexpensive. These precursors offer a sustainable pathway to CQD synthesis, which is in line with the circular economy and less petrochemical resource-dependent (Salimi Shahraki et al. 2022). The heteroatoms and functional groups present in these precursors are responsible for the improved optical properties and biocompatibility of the resulting CQDs, making them especially well-suited for sensitive, eco-friendly sensing platforms (Thakur et al. 2024). Thus, the judicious choice of carbohydrate source not only sustains the green nature of CDCQDs but also optimizes their performance for future analytical tools (Khan et al. 2023).

### Synthesis techniques

Synthesis of CDCQDs has evolved with a series of advanced, eco-friendly, and scalable processes each imparting a variety of different structural and photonic properties on the resulting nanomaterial. Hydrothermal and solvothermal processes are among the most widespread, where high-temperature treated (typically 150–250 °C) carbohydrate precursors are processed within aqueous or organic solvents under sealed autoclaves (John et al. 2023; Kasinathan et al. 2022; Kaur et al. 2024). These methods are lauded for their simplicity, scalability, and amenability to a broad array of natural carbohydrate precursors, allowing for effective one-pot carbonization and surface functionalization.

Microwave-assisted synthesis is a major advance, allowing rapid and homogenous heating with uniform speed that permits homogeneous nucleation and growth of CDCQDs. Reaction time is greatly reduced-often to less than ten minutes-while particle size distribution and fluorescent characteristics are maintained highly controllable. Pyrolysis and combustion processes, involving thermal degradation under inert or oxygen-starved atmospheres, produce graphitized CQDs with high fluorescence as well as increased electrical conductivity and are immediately ready for use where conductive nanomaterials are required (Kaur et al. 2022; Keerthana et al. 2023; Korram et al. 2023).

One of the glaring trends is the application of green synthesis strategies, where nontoxic solvents such as water or ethanol, removal of toxic reagents, and mild reaction conditions are employed as first priorities. Many of these sustainable protocols involve using plant extracts or dopants of natural origin, which are consistent with sustainability standards as well as enabling applications in biomedicine and environmental sensing. The choice of precursor and synthesis conditions plays a critical role in determining the QY, emission wavelength, and surface chemistry of CDCQDs, allowing for optimization for best use in a specific analytical requirement (Kumari et al. 2024; Liu et al. 2023a; Lo Bello et al. 2022). In general, these advances in synthesis enable the variety of applications and growing impact of CDCQDs in analytical chemistry, with further research directed toward improving reproducibility, scalability, and compatibility with emerging sensing platforms.

Hydrothermal/solvothermal synthesis remains the most widely used method for CDCQDs, offering flexibility in precursor choice and effective in-situ functionalization. However, long reaction times (several hours) and variability across batches hinder large-scale translation. By contrast, microwave-assisted synthesis dramatically reduces reaction time to minutes, often achieving comparable or higher QY due to uniform nucleation and growth. While limited by smaller batch volumes, microwave methods are energy-efficient and highly suitable for rapid, reproducible production. Together, these methods provide complementary strengths: hydrothermal for versatility and functionalization, microwave-assisted for speed and scalability, guiding researchers toward method selection based on application needs. In terms of energy efficiency, microwave-assisted synthesis is generally superior because it utilizes rapid and uniform dielectric heating, which reduces overall energy consumption and processing time compared to hydrothermal methods. However, while hydrothermal/solvothermal approaches are slower and require prolonged heating, they allow for larger batch volumes and better scalability.

### Surface functionalization and doping

Surface engineering of CDCQDs plays a crucial role in optimizing their optical, electronic, and sensing properties. Heteroatom doping with nitrogen, sulfur, and phosphorus enables intentional control of the electronic structure of the quantum dots to enhance photoluminescence, charge separation, and incorporation of redox-active sites critical to analytical performance (Lou et al. 2021; Luo et al. 2024). Nitrogen doping, for instance, increases electron density and QY, and sulfur and phosphorus can control redox potential and facilitate charge transfer processes.

Surface passivation, typically achieved by using small molecules such as polyethylene glycol, citric acid, or amino acids, easily reduces surface defects and yet improves QY and photostability. Even beyond simple passivation, biomolecule functionalization-with such as aptamers, DNA, or high-specificity ligands-includes considerable selectivity and sensitivity, enabling the construction of biospecific sensors with designed specificity for a range of analytes (Mehta et al. 2021). The interplay between analytical mechanism and surface chemistry-including fluorescence quenching, Förster resonance energy transfer (FRET), or turn-on/turn-off signaling-is crucial in defining the specificity and sensitivity of CDCQD-based sensors. Coupled with advanced surface modification methods are key to unleashing the full potential of CDCQDs for next-generation analytical and diagnostic platforms.

### Physicochemical properties of CDCQDs

Physicochemical characteristics of CDCQDs define their practical application and analytical performance. Morphologically, CDCQDs are typically quasi-spherical nanoparticles with diameters ranging from 2 to 10 nm, for which size uniformity is crucial for reproducible and consistent fluorescence emission. Optically, they exhibit intense ultraviolet–visible (UV–Vis) absorption bands primarily in the 250–350 nm region due to π–π* transitions of sp^2^-hybridized carbon atoms and n–π* transitions of surface groups. Photoluminescence response is excitation-independent or -dependent and primarily governed by synthesis pathways and surface oxidation degrees (Ma et al. 2023; Manjubaashini et al. 2024). QY vary widely, inclined to range from 5% to as much as 70%, depending on precursors selected, synthesis conditions, and passivation treatments at the surface.

Surface chemistry plays a pivotal role in the function of CDCQDs; several oxygen- and nitrogen-rich functional groups such as hydroxyl (–OH), carboxyl (–COOH), amino (–NH₂), and carbonyl (C=O) units offer effective aqueous solubility, colloidal stability, and facilitate selective molecular recognition. The functional groups also facilitate facile surface engineering, which enhances biocompatibility and enables target-specific interactions in sensing platforms. Besides, CDCQDs display very good stability under a broad range of pH conditions (3–10), against varying ionic strengths, and under prolonged illumination, favoring their endurance for real analytical use. Overall, these physicochemical features render CDCQDs highly suitable for sensitive, selective, and reproducible sensing in biomedical diagnostics, environmental monitoring, and food safety authentication (Raja et al. 2021; Raja et al. 2024).

### Comparison with conventional sensors

Compared to conventional sensing platforms such as organic dyes, metal nanoparticles, or semiconductor-based probes, CDCQDs offer distinct advantages and some drawbacks. Their cost is generally lower, as they can be synthesized from inexpensive carbohydrate-rich wastes; however, post-synthesis functionalization can increase expenses. In terms of energy consumption, microwave- and hydrothermal-based CDCQD synthesis is more sustainable than the high-temperature or vacuum deposition processes used for semiconductor sensors. Lifespan and stability also favor CDCQDs, as they exhibit high photostability and can be reused multiple times without significant loss of performance, unlike dyes that photobleach rapidly. On the other hand, conventional sensors currently enjoy higher industrial standardization and regulatory acceptance, meaning CDCQDs must still overcome reproducibility and scale-up challenges to fully establish their competitive edge.

To overcome current scalability challenges, interdisciplinary collaborations will be essential. Integration of artificial intelligence (AI) and machine learning can support smart signal processing, multiplexed analyte detection, and real-time data analytics for CDCQD-based sensors, enabling their use in complex environmental or biomedical samples. Partnerships with chemical engineers and materials scientists could accelerate the design of continuous-flow or pilot-scale reactors for reproducible, high-yield CDCQD production. Collaboration with industrial partners in food safety, clinical diagnostics, and environmental monitoring would further validate performance under real-world conditions. Such pilot-scale studies, coupled with digital integration and device miniaturization, represent a practical pathway to bridge the gap between laboratory innovation and industrial adoption.

## Analytical chemistry applications

### Chemical sensing

Carbohydrate-source carbon quantum dots (CDCQDs) have rapidly gained prominence as a revolutionary set of nanomaterials for chemical sensing, owing to their excellent optical properties, biocompatibility, and green synthesis (Raju et al. 2024; Ren et al. 2025; Šafranko et al. 2021). The nanodots, typically less than 10 nm in diameter, are synthesized from a vast array of carbohydrate sources including mono-, di-, and polysaccharides, and biomass waste. Their rich carbonyl and hydroxyl surfaces promote simple doping and surface modification that, in return, modulates their fluorescence character and enhances their sensitivity and specificity toward target analytes.

CDCQDs function primarily by mechanisms based on fluorescence such as static and dynamic quenching, fluorescence enhancement or turn-on, and reversible "on–off-on" signaling. The mechanism chosen depends on the analyte and the type of surface modifications added to the CQDs (Raja et al. 2024). For instance, electron-donating or -withdrawing groups and heteroatom doping (e.g., nitrogen and sulfur) strongly influence the photoluminescence response, thereby providing a rich platform for sensing a vast array of chemical species (Table 1).

**Table 1:** Comparison of Hydrothermal/Solvothermal and Microwave-Assisted Synthesis of CDCQDs

Method	Reaction Temperature (°C)	Reaction Time	Typical Yield	QY Range	Advantages	Limitations
Hydrothermal /Solvothermal	150–250	4–12 h	Moderate (30–60%)	10–50%	Simple setup, scalable, good surface functionalization	Long reaction time, batch-to-batch variation
Microwave-assisted	120–200	5–20 min	Moderate–High (40–70%)	15–60%	Rapid, uniform heating, energy-efficient, controllable particle size	Limited batch volume, specialized equipment

**Fig. 1:**
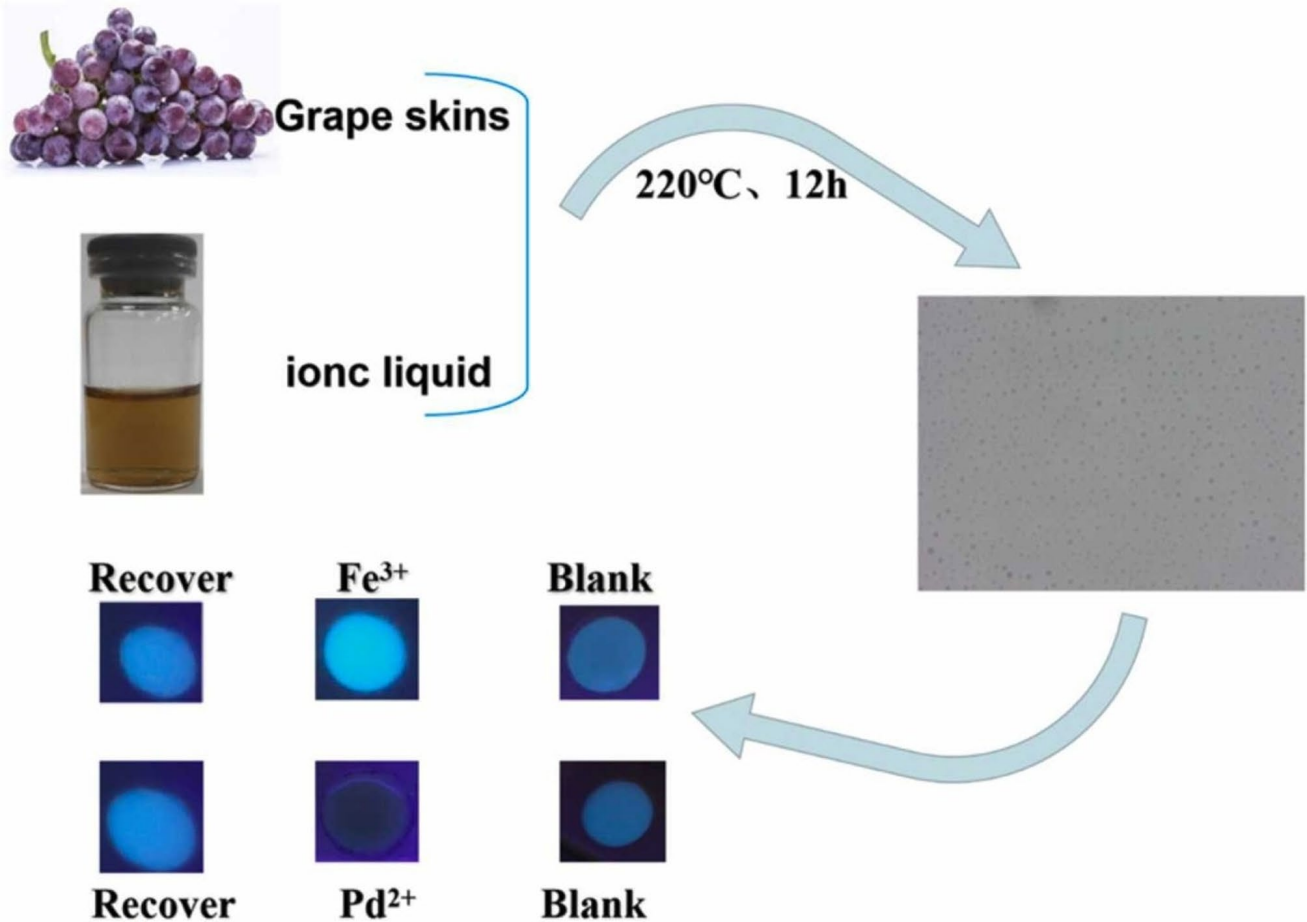
Ionic liquid modified biomass for the detection of Fe^3+^ and Pd^2+^ in environmental water (Kang et al. 2023)

The sensitivity and selectivity of CDCQDs are directly linked with their surface chemistry. For example, ionic liquid-functionalized CQDs from grape skin have facilitated record detection limits for Fe^3^⁺ ions down to 0.001 nM (Kang et al. 2023) (Fig. 1). The ionic liquid offers superior surface passivation and efficient energy transfer, resulting in bright and stable fluorescence and minimal background interference. These state-of-the-art surface engineering strategies demonstrate the potential of CDCQDs for ultra-trace analysis in complex matrices. On the other hand, CQDs synthesized from simpler carbohydrate precursors, such as Borassus flabellifer endosperm, offer simpler synthesis but less sensitive detection limits (e.g., 2.01 µM for Fe^3^⁺) (Nagaraj et al. 2022). While still usable, such systems highlight the necessity of precursor selection and post-synthetic modification in achieving optimal analytical performance (Table 2).

**Table 2:** Comparative analysis of heavy metal ion detection using CDCQDs

Name and source	Target ion and detection limit	References
ILB-CQDs from grape skin (modified with C_2_H_4_O_3_:C_5_H_9_NO_2_ ionic liquid)	Fe^3^⁺: 0.001 nM; Pd^2^⁺: 0.23 µM	Kang et al. (2023)
CQDs from endosperm of Borassus flabellifer	Fe^3^⁺: 2.01 µM	Nagaraj et al. (2022)
N,S-CDs from chitosan and κ-carrageenan	Fe^3^⁺: 57 nM. Ascorbic acid (AA): LOD = 38 nM	Xu et al. (2021)
Lignin carbon dots	Fe^3+^: 69 μmol/L	Du Peng et al. (2023)
Pumpkin seeds	Cu^2+^ and Fe^3+^ ions were 0.43 and 0.11 μM	Jeeva et al. (2024)
Walnut green skin	Pb^2+^: 1.55 nmol/L	Xing et al. (2024)
Carboxymethyl cellulose	Cu^2+^: 3.42 μM	Liang et al. (2024a)
Cellulose	Fe^3+^: 0.15 μM	Passos Zattar et al. (2022)
Lemon peel	Cu^2+^: 0.43 μM	Su et al. (2024)
Canon ball	Fe^3+^: 0.071 μM	Ashok Varman et al. (2022)
Sugarcane waste	Hg^2+^: 0.1 μM	Kasinathan et al. (2022)
Cellulose	Cu^2+^: 23.4 nM	Wang et al. (2022a)
Sodium alginate	Hg^2+^: 2 ppb (0.01 μM)	Thakuri et al. (2024)
Maple leaves	Cesium ions: 160 nM	Chellasamy et al. (2022)
Mexican Mint extract	Fe^3+^: 53µM	Architha et al. (2021)
Grapefruit juice	Cr(VI): 0.155 μM	Wang et al. (2022b)
Bamboo-derived cellulose	Hg^2+^: 5.16 nM	Bavya et al. (2025)
Lignin	Fe^3+^: 0.77 μM	Zhu et al. (2021a)
Discarded cigarette butts	Fe^3+^: 0.2 μM	Yang et al. (2024)
Mopan persimmons	Fe^3+^: 0.324 μM	Ma et al. (2023)
Poa pratensis	Mn^2+^ and Fe^3+^ ions are 1.2 and 1.4 μM	Krishnaiah et al. (2022)
Myrrh residue	Fe^3+^: 24.62 μM	Huang et al. (2023)
Aspergillus niger	Cr^6+^, Fe^3+^, and Co^2+^	Loa et al. (2023)
Peanut shell powder	Fe^3+^: 2.768 μM	Yang et al. (2025)
Prosopis juliflora	Fe^3+^: 593 nM	Kirubanithy et al. (2025)
Elephant manure	Hg^2+^, Fe^2+^, and Fe^3+^ were 1.03, 18.52, and 0.65 μM	Seesuea et al. (2024)
Caryocar coriaceum	Fe^3+^: 1.16 μmol/L	Oliveira et al. (2025)
Pumpkin	Cd^2+^: 0.68 μmol/L	Liao et al. (2024)
Faba bean seeds	Hg^2+^: 0.35 μM	Fallah et al. (2023)
Jengkol peels	Hg^2+^: 0.03 μM	Prayugo et al. (2023)
Hakka yellow wine lees	Fe^3+^: 0.18 μg/mL	Yu et al. (2024)
Ganoderma lucidum bran	Cu^2+^: 0.74 μmol/L	Wang et al. (2023a)
Beetroot fruit (Beta Vulgaris) bagasse	Cu^2+^ and Ag^+^	Rojas-Valencia et al. (2023)
Lycium barbarum	Cr^6+^: 0.16 µmol/L	Xie et al. (2024)
Pine cone	Cu^2+^: 0.005 μg/mL	Sanni et al. (2022))

#### Heteroatom doping

The addition of heteroatoms such as nitrogen and sulfur further adds to the flexibility of CDCQDs. Chitosan- and κ-carrageenan-based N,S-co-doped CQDs are a case in point, and they represent a case of dual-analyte detection. The dots can successively (via fluorescence quenching) and selectively (via fluorescence recovery) detect Fe^3^⁺ and ascorbic acid with detection limits of 57 nM and 38 nM, respectively (Xu et al. 2021). Their reversible fluorescent behavior, which is generated by static aggregation and electron transfer, renders them particularly valuable for real-time sequential sensing applications.

Heteroatom doping, particularly with nitrogen, sulfur, and phosphorus, plays a decisive role in modulating the electronic and optical properties of CDCQDs. Beyond general improvements in QY and redox activity, mechanistic insights reveal that the configuration of doped sites dictates performance. For example, graphitic N sites contribute to enhanced electron density and electrical conductivity by delocalizing π-electrons across the carbon framework, thereby improving charge transport. In contrast, pyridinic N sites introduce localized lone pairs that serve as active coordination centers, enabling stronger binding interactions with analytes and promoting fluorescence quenching or recovery processes. Sulfur doping typically introduces electron-donating thiophene-like sites, modulating redox potential, while phosphorus doping induces electron-rich domains that shift bandgaps and facilitate charge separation. Collectively, these dopant-specific mechanisms explain why N,S,P co-doped CDCQDs often demonstrate superior sensitivity, selectivity, and stability in analytical applications.

#### Effect of functional groups

In addition to metal ion detection, CDCQDs have been designed to contain intrinsic antioxidant activity. For example, CQDs made from table sugar and glutathione (GSH) not only detect Hg^2^⁺, Fe^2^⁺, Fe^3^⁺, and As^5^⁺ ions via thiol coordination and charge transfer but also show extensive DPPH radical scavenging activity (Chobpattana et al. 2025). This dual functionality further enhances their utility, enabling the simultaneous sensing and reduction of oxidative stress in environmental and biological samples.

The preservation of some functional groups during synthesis can impart CDCQDs with unique spectral characteristics. ChlCQDs derived from banana leaf extract with retained chlorophyll moieties fluoresce in the red part of the spectrum (~ 660 nm) and have differential affinities for arsenic and mercury ions (Bayazeed Alam et al. 2022). Fluorescence of the dots is enhanced by As^3^⁺ and quenched by Hg^2^⁺, enabling a "turn-off/turn-on" detection strategy. Density Functional Theory (DFT) calculations validate the selectivity, with strong binding interactions between target ions and chlorophyll functionalities.

#### Composites and hydrogels

More recent advances have included CDCQDs in composite materials and hydrogels, extending their range of applications even further. Supramolecular composites, such as poly(β-cyclodextrin-co-citric acid) containing CQDs, are capable of detecting multiple divalent metal ions (e.g., Ni^2^⁺, Cu^2^⁺, Cd^2^⁺, Pb^2^⁺) with large Stern–Volmer constants, indicating high sensitivity. They also serve as adsorbents, with metal removal efficiencies > 90% and exhibiting good recyclability (Silva et al. 2024). Hydrogel-based CDCQD systems, which are formulated from glycerol-starch-citric acid-chitosan–gelatin mixtures, exhibit very high QY (up to 83%) and superior mechanical strengths. Hydrogels exhibit superior sensitivity to Fe^2^⁺, Fe^3^⁺, and Hg^2^⁺ in aqueous solutions and are well-adapted for real-time environmental monitoring applications (Hassanzadeh Baraz et al. 2024).

#### Using in heavy metal removal

CDCQDs have found greatest utility for environmental applications, such as the detection of chromium (Cr(VI)) at levels below World Health Organization (WHO) regulatory tolerance levels. Nitrogen-doped CQD/poly(AMPS) hydrogel composite materials, for instance, offer detection limits of as little as 5.3 µg/L with recyclability and excellent adsorption capability. These features are of critical concern to the application of CDCQD-based sensors to regulatory compliance and environmental remediation processes (Tohamy 2024).

The integration of CDCQDs into visual and handheld detection formats is emphasized in the example of MAA films composed of Mesua Ferrea-derived CQDs. These films enable rapid (less than 5 min) and highly sensitive Fe^3^⁺ ion detection with limits of detection as low as 0.005 ppm. The functioning mechanism, as indicated by fluorescence lifetime analysis and Stern–Volmer plots, is dynamic quenching, which provides speed and field amenability (Baruah et al. 2025).

The CDCQD-based field of chemical sensing is characterized by sheer innovation and diversity. By judicious selection of carbohydrate precursors, planned surface modification, and fabrication of multifunctional composites, CDCQDs have evolved ultra-sensitive, selective, and often recyclable sensing platforms. Their ability to detect a broad spectrum of analytes-ranging from heavy metals and small biomolecules to environmental toxins-placing them in the pole positions of analytical devices of the future.

The continued innovation in green synthesis methods, mass-scalable production, and seamless integration with digital technologies (e.g., smartphone-readable outputs, microfluidics) will be the focus of bringing CDCQDs from the laboratory to the world. With solving the problems of reproducibility and regulatory approval, CDCQDs will come to be a part of environmental monitoring, clinical diagnosis, and monitoring of food safety, a typical confluence of sustainability, sensitivity, and specificity.

While CDCQDs exhibit outstanding sensitivity, particularly at sub-nanomolar levels, a critical trade-off often arises between sensitivity and selectivity. In complex matrices, structurally or chemically similar ions such as Fe^3^⁺ and Cu^2^⁺ may produce overlapping fluorescence responses due to comparable redox potentials and coordination chemistry. This cross-reactivity can compromise selectivity, even when detection limits are extremely low. Surface functionalization strategies—such as introducing specific ligands, aptamers, or heteroatom dopants—are therefore essential to discriminate between competing analytes. Balancing high sensitivity with robust selectivity remains a central challenge, and future research must emphasize designing CDCQDs that minimize false positives while maintaining ultrasensitive detection thresholds.

CDCQDs have demonstrated unrivaled capability as selective and sensitive fluorescent probes for the determination of a wide range of toxic organic compounds such as pesticides, herbicides, and dyes. Their synthesis from renewable biomass feedstocks such as vegetables, fruits, and agricultural wastes not only highlights their environmental compatibility but also imparts unique surface chemistries that improve their analytical performance (Table 3).

**Table 3:** Comparative analysis of pesticide, herbicides, and dyes detection using carbohydrate-based CQDs

Name and source	Targeted analyte and detection limit	References
Cauliflower-derived CDs	Diazinon – 0.25 ng/mL; Amicarbazone – 0.5 ng/mL; Glyphosate – 2 ng/mL	Ashrafi Tafreshi, et al. (2020)
CDs from Greengage juice	Pesticides (dialen super, duplosan, confidor). 10⁻⁸ ppm	Alvandi et al. (2021)
Mulberry leaves (cellulose-rich)	Parathion-methyl (MP): 0.14 µM; Glyphosate: 0.60 µM	Yang et al. (2023)
Waste Tea-derived CDs	Quinalphos 25 EC – 0.2 ng/mL; Thiamethoxam 25 WG – 1 ng/mL Propargite 57 EC – 10 ng/mL	Sinha and Ray (2024)
Lemon Peel-derived CDs (LPCDs)	Fe^3^⁺ – 0.18 µM; Propiconazole (PC) – 0.054 µM	Vadia et al. (2023)
CdTe–CQD integrated probe (CQDs from chitosan)	Glyphosate = 2 pM	Bera and Mohapatra (2020)
Rambutan seed waste N-CQDs	Congo Red (CR): 0.035 µM (≈11.2 ppb)	Zulfajri et al. (2023)
Impatiens leaf dual-emissive CDs	Methylene Blue (MB), 15 nM (≈0.005 ppb)	Guo et al. (2025)
Water spinach-derived CQDs	Crystal Violet (CV), 710 nM (≈0.23 ppm)	Yin et al. (2024)
Olive pomace-derived CDs (WP-CDs)	Methyl Orange (MO), 151 ppb (≈0.46 µM)	Sousa et al. (2022a)
Cellulose-derived CQDs	Allura Red (AR) and Ponceau 4R (PR) food dyes; AR: 0.45 μg/mL; PR: 0.47 μg/mL	Gunjal et al. (2021)
Olive-waste CD	Methyl Orange, 151 ppb (0.46 μM)	Sousa et al. (2022b)
N,S-Doped CQDs (from Giloy plant stem – a starch-rich herbal source)	Congo Red (CR) 0.062 μM; 4-Nitrophenol in same study) 62 nM	Swain and Jena (2025)
“Green” CQDs from cauliflower (vegetable juice, rich in cellulose)	Pesticides: Diazinon (0.25 ng/mL), Amicarbazone (0.5 ng/mL), Glyphosate (2 ng/mL)	Ashrafi Tafreshi et al. (2020)
N-doped CQDs from chitosan (polysaccharide) plus 4-hydroxycoumarin	Antibiotics: Tetracycline (120 μM), Oxytetracycline (127 μM), Chlortetracycline (117 μM)	Liu, et al. (2025)
Water-soluble CDs from microcrystalline cellulose (pure cellulose hydrothermal carbonization)	Antibiotic: Ofloxacin (fluoroquinolone) ≈0.025 ppm (~ 0.07 μM)	Aggarwal et al. (2023)
Ca, N, S co-doped CDs from Miswak plant root (a cellulose-rich medicinal root) with m-phenylenediamine	Dye: Congo Red (azo dye) – linear range 0.2–1.2 μM, LOD 58 nM	Durrani, et al. (2022)
Glucose-Derived CQDs	Mesotrione ~ 0.054 μg/mL	Rani et al. (2023)
Starch-Derived CQDs	Fe^3^⁺ for 0.024 μM and glyphosate for 0.036 μM	Liang et al. (2024b)
Fruit shells	Tyramine in fermented meat products: 0.77 µg/L	Zhang et al. (2024a)
Walnut Shell	Acebrophylline, 0.142 nM	Perumal et al. (2025)
Lycium ruthenicum	Sunset Yellow, 17 nM	Zhang et al. (2024b)
Pomegranate peel	Kojic acid in food, 30 ± 0.04 µM	Hassan et al. (2024)
Banana peel	Resveratrol in food, 2.21 ng/mL	Wang et al. (2025)
Tetraclinis articulata extract	Tizanidine in human urine and dosage forms; 0.024 μg/mL	Elhak et al. (2025)
Agricultural waste	Ciprofloxacin in human blood serum and urine samples, 21.3 nM	Sadeghi-chahnasir et al. (2025)
Tobacco leaves	Tetracycline, 0.52 μM	Liang et al. (2022)
Wild lemon (Citrus pennivesiculata)	Tetracycline, 0.42 µM	Venugopalan and Vidya (2023)
Boerhavia diffusa	Methyl parathion (MP) pesticide and uranyl ions (UO_2_^2+^), 22.4 nM and 4.4 nM	Renu,, et al. (2025)
Crocus cancellatus	Ponceau 4R dye in food, 40 nM	Esmail and Jabbar (2023)
Aegle Marmelos	Brilliant green dye and Cu^2+^, -	Rani and Shanker (2024)
Cabbage and onions	Nitazoxanide drug, 0.07 μM	Qandeel et al. (2023)
Miswak (Salvadora persica)	Congo red, 58 nM	Durrani et al. (2022)
Lotus seed plumules	Folic acid, 0.27 μM	Liu (2025)
Flammulina velutipes	Hydroxyl radical, 95 nM	Hou et al. (2023)
Bean shell	Malachite green in food, 0.07 μM	Wang et al. (2024)
Sucrose	Torsemide in its tablets and plasma samples, 0.027 μg/mL	Abo Zaid et al. (2024)
Passion fruit shells	Tyramine in fermented meat products, 1.3 μg/kg	Zhang et al. (2024c)
Gandha Prasarini	Tartrazine, 0.18 μM	Mohanta et al. (2023)
Cellulose	Nicosulfuron, 0.082 μM	Liu et al. (2025)

**Fig. 2:**
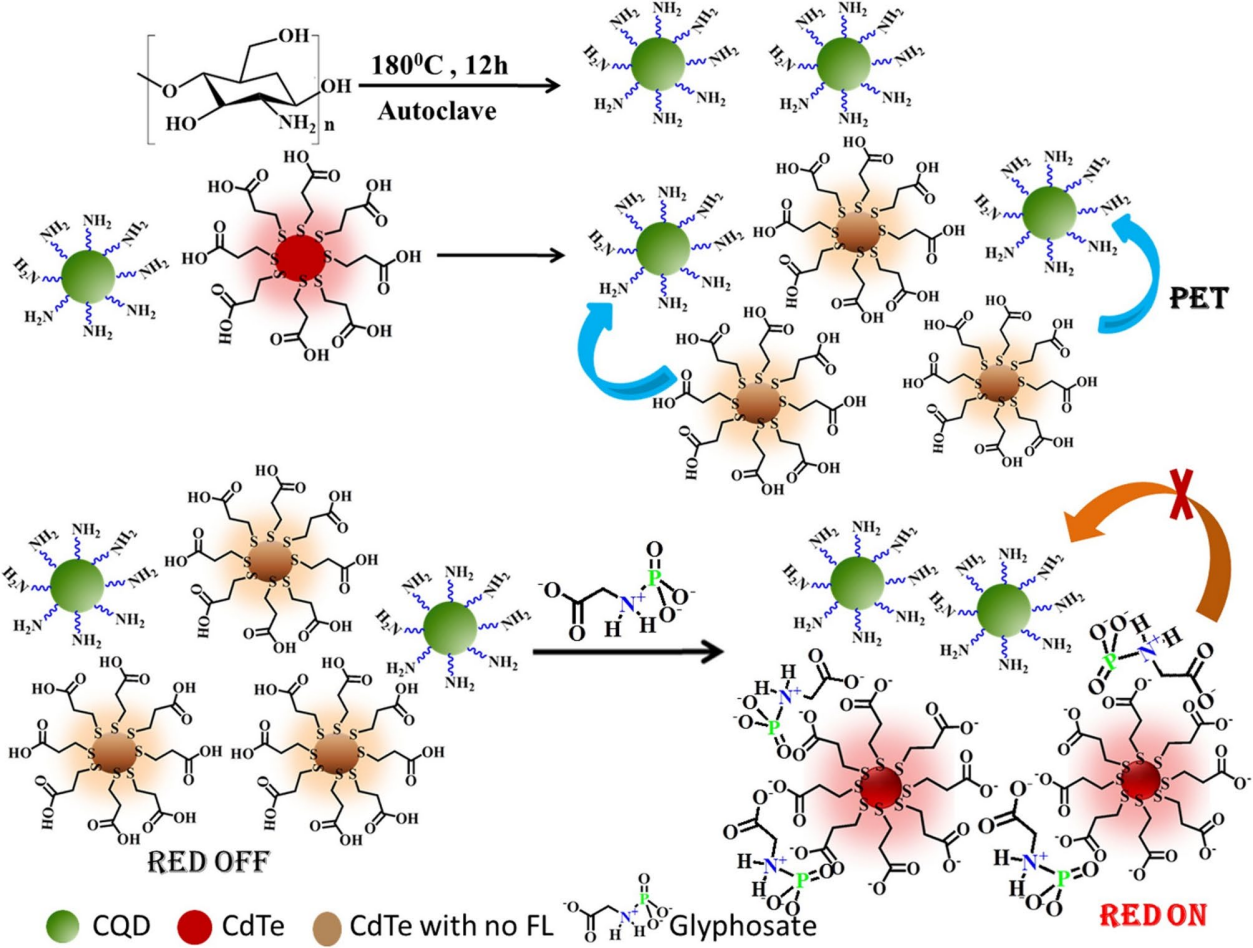
Illustration of sensing mechanism of CQD-CdTe chemosensor towards glyphosate (Bera and Mohapatra 2020)

CDCQDs possess different fluorescence-based sensing mechanisms-including fluorescence quenching, turn-on/turn-off responses, inner filter effects (IFE), and fluorescence resonance energy transfer (FRET)-that enable ultra-low limits of detection (LODs) typically achieving nanomolar or even picomolar levels. For example, cauliflower-derived CQDs were highly sensitive toward pesticides with LODs of 0.25 ng/mL toward diazinon, 0.5 ng/mL toward amicarbazone, and 2 ng/mL toward glyphosate, based on fluorescence quenching with quick response rates and good selectivity that was independent of structurally unrelated pesticides (Ashrafi Tafreshi, et al. 2020). Similarly, chitosan-derived CdTe–CQDs showed an ultra-sensitive LOD of 2 pM for glyphosate via an inhibition of photoelectron transfer turn-on fluorescence mechanism, though these sensors were one time only (Fig. 2) (Bera and Mohapatra 2020). It outlines the development of a CdTe–CQD fluorescence "turn-on" sensor for the rapid and sensitive detection of glyphosate in aqueous media. Utilizing a photoinduced electron transfer (PET) mechanism, the fluorescence of CdTe at 619 nm is quenched by CQDs and restored upon glyphosate addition due to disintegration of the CdTe–CQD complex. The sensor exhibits a broad linear detection range (0–1000 nM) with an exceptionally low detection limit of 2 pM and demonstrates high selectivity against other organophosphorus compounds. Its simple design, low cost, and successful application in real vegetable samples make it a promising tool for environmental and food safety monitoring.

Other CDCQDs based on biomass have shown pesticide sensing across a broad spectrum. Greengage juice-based CQDs sensed pesticides such as dialen super and confidor at 10⁻⁸ ppm via the application of fluorescence quenching to render high sensitivity (Alvandi et al. 2021). Mulberry leaf-extracted CQDs utilized dual-emission at 440 nm and 660 nm to detect parathion-methyl (0.14 µM) and glyphosate (0.60 µM) by inner filter effects and Cu^2^⁺-assisted static quenching, respectively (Yang et al. 2023). Waste tea-extracted CQDs utilized a FRET-based turn-on/off mechanism to detect quinalphos (0.2 ng/mL), thiamethoxam (1 ng/mL), and propargite (10 ng/mL) with satisfactory selectivity and recyclability even in complex pesticide mixtures (Sinha and Ray 2024).

Likewise, lemon peel-derived CQDs (LPCDs) were able to detect Fe^3^⁺ (0.18 µM) and the fungicide propiconazole (0.054 µM) using a reversible fluorescence “turn-off/on” response. These sensors were applicable in pharmaceutical and vegetable samples and were recyclable through the use of cellulose paper strips (Vadia et al. 2023).

In the detection of herbicides, CQDs based on starch exhibited high selectivity towards metal ions for the detection of glyphosate (0.036 µM) and Fe^3^⁺ (0.024 µM) (Liang et al. 2024b). Glucose-based CQDs also demonstrated good applicability by detecting mesotrione at 0.054 µg/mL with recovery rates between 95 and 105% in food and soil samples (Rani et al. 2023).

CDCQDs have also proved to be effective in the detection of toxic dyes with high selectivity and sensitivity. For instance, nitrogen-doped CQDs obtained from rambutan seeds detected Congo Red at 0.035 µM with maintaining selectivity in tap and lake water matrices (Zulfajri et al. 2023). Dual-emissive CQDs synthesized from leaves of Impatiens balsamina enabled ratiometric fluorescence detection of methylene blue at 15 nM, where red emission was quenched while blue emission remained constant, enabling selective detection in water and food samples (Guo et al. 2025). Water spinach-derived CQDs sensed crystal violet at 710 nM through fluorescence quenching with excellent selectivity in aquaculture samples (Yin et al. 2024).

**Fig. 3:**
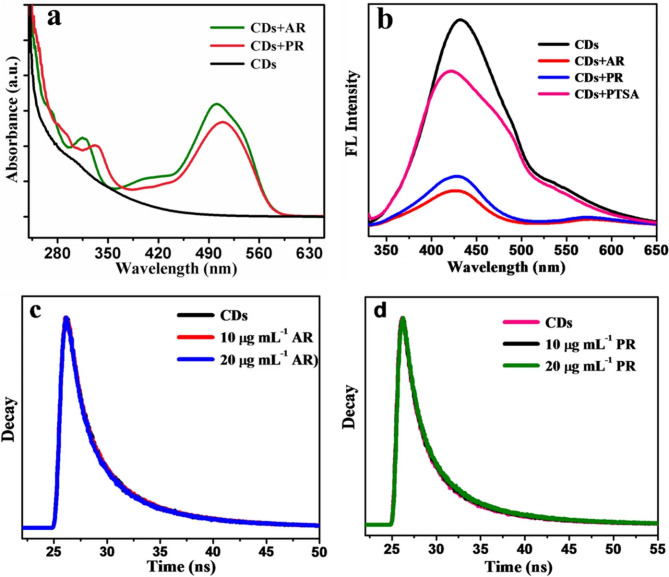
Interpretation of mechanism of quenching through; **a** Absorption spectrum **b** fluorescence spectrum; life time spectrum with AR dye (**c**) and with PR dyes (**d**) (Gunjal et al. 2021)

Olive pomace- and olive-waste-derived CQDs effectively detected methyl orange at 151 ppb (~ 0.46 µM) by static quenching of IFE with signal stabilization within two minutes and with high selectivity against other dyes such as methyl red and rhodamine (Sousa et al. 2022a, 2022b). Cellulose-derived CQDs also detected food dyes Allura Red and Ponceau 4R with LODs of 0.45 and 0.47 µg/mL, respectively, validating their efficacy in actual soft drink samples (Gunjal et al. 2021). Figure 3 explains the fluorescence quenching mechanism of CDs upon interaction with Allura Red (AR) and Ponceau 4R (PR) dyes. The absorption spectra (Fig. 3a) show diminished high peaks of CDs at 300 nm after dye addition, suggesting ground-state complex formation. Fluorescence spectra (Fig. 3b) further confirm this through a noticeable blue shift, implying strong interactions between the sulfonic acid groups of the dyes and the carboxyl/hydroxyl groups on the CDs. This interaction was validated using para-toluene sulfonic acid (PTSA), which caused similar quenching. Finally, time-resolved fluorescence decay profiles (Fig. 3c, d) reveal no significant changes in the lifetime of CDs upon dye addition, confirming that the quenching mechanism follows a static (rather than dynamic) pathway driven by ground-state complexation.

The CQDs from stem of Giloy plant combined smartphone-assisted colorimetric sensing of Congo Red with 95–99% recovery in real water samples, adding sensor portability and accessibility (Swain and Jena 2025). Most of the CDCQD-based sensors are reusability designed, such as tea waste, cauliflower, and lemon peel-derived sensors, which makes them more cost-effective and sustainable. Response times tend to be short, typically ranging from 1–2 min, such that these sensors are able to carry out real-time detection under field conditions. Surface functionalization routes-e.g., doping with sulfur, nitrogen, and amino acids-and spectral compensation (e.g., red-shifted fluorescence or dual-emission) are instrumental in their high sensitivity and selectivity.

Overall, CDCQDs represent an extremely promising nanomaterial family for environmental and food safety surveillance. Their very low detection thresholds, fast response, high selectivity, and potential recyclability render them as ideal candidates to be incorporated in portable, field-deployable sensing devices. Future research ought to target multiplexed detectability and further compatibility with smartphone-based analytical equipment and smart package systems in order to widen their practical utilization.

### Biomedical diagnostics

CDCQDs are a type of high-performance and multifunctional nanomaterial that has been applied in biomedical diagnostics. Due to their compatibility, fluorescence, and surface functionalization, CDCQDs are utilized in the sensitive, selective, and multiplexed sensing of clinically important biomolecules. The current chapter is a thorough review of the latest progress in the utilization of CDCQDs for the sensing of antibiotics, glucose, amino acids, vitamins, and other biomarkers and their incorporation into point-of-care diagnostic devices (Table 4).

**Table 4:** Comparative analysis of biological objects detection using carbohydrate-based CQDs

Name and source	Analytes and detection limit	References
N,S-co-doped biomass-based CDs (C-dots)	Tetracycline antibiotics	Yang et al. (2022)
CMC-CQDs nanocomposite (Steglich esterification-based)	Glucose, LOD = 7 nM	Patra et al. (2024)
Gelatin-derived Carbon Quantum Dots (CQDs)	Paraquat-induced neurotoxicity (protective against ROS elevation, neuronal damage)	Ahlawat et al. (2022)
Glucose (with pyridine doping) Pyridine-Functionalized CDs	l-Tryptophan (Trp, amino acid); Human serum; live cells. LOD 5.7 nM	Li et al. (2020)
Chitosan-CQD Impedance Aptasensor for Sialic Acid	Sialic acid (neuraminic acid). Human serum (spiked)	Elgendi et al. (2024)
Glucose + boric acid (B-doped CDs)	Glucose, Human serum; live cells. LOD ~ 10 µM	Yu et al. (2022)
Potato Starch N-doped CQDs	Ascorbic acid (Vitamin C). LOD 0.093 µM; linear 0–130 µM	Preethi et al. (2022)
Zinc gluconate (gluconic acid) CQD	Riboflavin (Vitamin B2)	Meng and Wu (2024)
Carrageenan/Lysine CQDs	Folic acid (Vitamin B9) ~ 40 nM	Paul, et al. (2022)
Carbon QDs in DCC-Assisted Cellulose	Glucose, Human blood (serum); 7 nM	Patra et al. (2024)
Chitosan-CQDs	Glucose, Human blood (serum), LOD 1.51 µM	Patra, et al. (2023)
Biomass (Pear juice (fruit sugars)) CQDs	Ascorbic acid (AA), Aqueous solution (with interferents). LOD 1.27 µM	Das et al. (2019)
Glucose-derived (N-doped)	Histidine (amino acid), Spiked urine sample	Liu et al. (2023b)
Sugar based Turn-Off/On CQDs	Cys, Hcy, GSH (thiols), Fetal bovine serum, LODs 70–110 nM	Shellaiah and Sun (2023)
Ginkgo kernels based CQDs	Nitrite (NO₂⁻) for 0.15 μmol/L	Zhang et al. (2022)

#### Glucose sensing

The use of QDs has significantly advanced the field of glucose detection, which is crucial for managing diabetes and monitoring metabolic processes. CMC-CQDs, which were created through Steglich esterification, have achieved a remarkable limit of detection (LOD) of 7 nM, with two distinct linear detection ranges (0–0.06 mM and 1.28–61.44 mM), and recovery rates of 100 ± 5%, even in challenging environments with high ionic strength and varying pH levels (Patra et al. 2024). Chitosan-based CQDs demonstrated a LOD of 1.51 µM, with a linear detection range up to 10 mM, and recovery rates in serum samples ranging from 95.8 to 107.3% (Patra et al. 2023). Additionally, intracellular glucose imaging with a LOD of approximately 10 µM was made possible by boron-doped glucose CDs, which used boronate affinity for specific glucose recognition (Yu et al. 2022). One alternative method, which used CQDs embedded in DCC-assisted cellulose, produced a LOD of 7 nM and near-quantitative recovery in human blood (Patra et al. 2024).

#### Amino acid and biomolecule detection

Detection of amino acids and their related biomolecules is important in clinical diagnostics and metabolic profiling. Pyridine-doped glucose CDs have facilitated the selective sensing of l-tryptophan through fluorescence quenching with an LOD of 5.7 nM in human serum and living cells, with outstanding selectivity and recovery rates in the range of 98–105% (Li et al. 2020). For histidine detection, glucose-derived N-doped CQDs utilized a dual-emission ratiometric fluorescence response, via Ni^2^⁺–dye displacement mediation, to achieve low-µM detection limits and superb specificity (Liu et al. 2023b). Thiol-selective CQD probes were also developed, which can detect cysteine (Cys), homocysteine (Hcy), and glutathione (GSH) on the basis of a turn-off/on mechanism (Hg^2^⁺ quenching reversed by thiol binding), with LODs of 70–110 nM and 96–105% recovery rates in serum (Shellaiah and Sun 2023).

#### Vitamin sensing

CDCQDs offer suitable platforms for detecting vitamins, which is crucial for clinical and nutritional research. Potato starch-derived N-CQDs have delivered the highly sensitive detection of ascorbic acid (vitamin C) through hydrogen bonding-induced aggregation with a LOD of 0.093 µM and near-quantitative recovery in tablets of supplements (Preethi et al. 2022). Zinc gluconate-derived CQDs measured vitamin B2 (riboflavin) by a static quenching process in an "on–off-on" fluorescence mode with an LOD of ~ 40 nM and 98–101% recovery yields in tablets, juices, and serum (Meng and Wu 2024). Carrageenan/lysine-derived CQDs selectively monitored folic acid (vitamin B9) in fortified foods, tablets, and wastewater with an LOD of ~ 40 nM and 95–105% recovery yields even with numerous potential interferents (Paul et al. 2022).

#### Point-of-care diagnostics and imaging applications

**Fig. 4:**
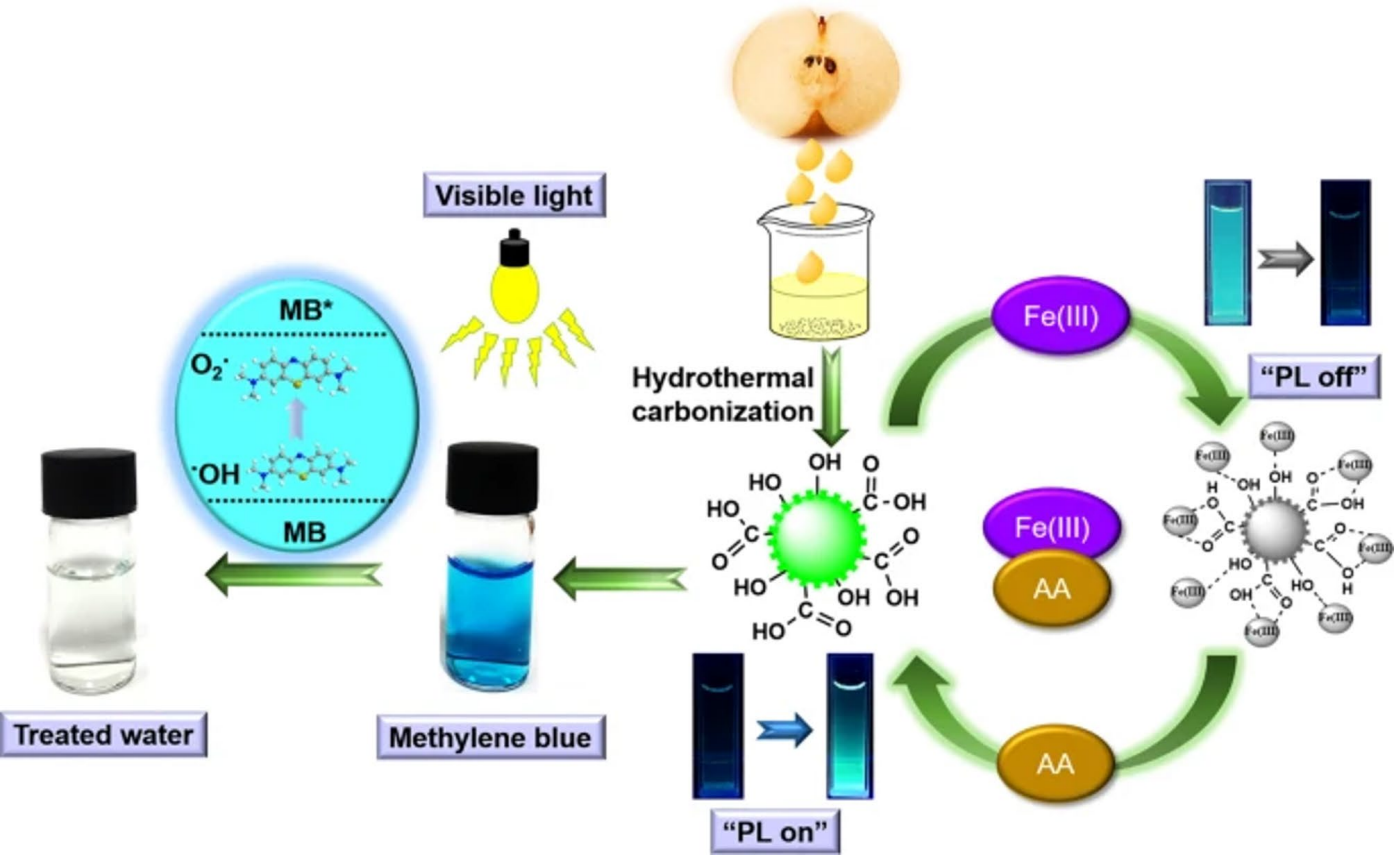
Schema of pear juice-derived CQDs in detection of Fe^3^⁺ and ascorbic acid (Das et al. 2019)

The use of CDCQDs in point-of-care diagnostic platforms is a rapidly developing field. For instance, an electrochemical aptasensor based on chitosan and CQDs allowed for the ultra-sensitive detection of sialic acid, a glycan biomarker linked to cancer, in serum with a detection in the low nanomolar range and high recovery (Elgendi et al. 2024). In addition to detection, CDCQDs have demonstrated therapeutic and imaging capabilities; CQDs derived from gelatin have shown neuroprotective effects against oxidative stress in neuronal cells and C. elegans (Ahlawat et al. 2022), and ginkgo kernel-derived CQDs (QY 37.8%) have been used for MCF7 cancer cell imaging and the detection of nitrite (LOD 0.15 µM) in processed foods (Zhang et al. 2022). Pear juice-derived CQDs enabled dual detection of Fe^3^⁺ and ascorbic acid, with a LOD of 1.27 µM for vitamin C and 99% recovery, demonstrating their versatility in biosensing and photocatalysis (Fig. 4) (Das et al. 2019).

Although CDCQDs routinely achieve high recovery rates (95–105%) in spiked samples, real-world matrices introduce complexities that can compromise accuracy. Humic acids in natural waters may strongly absorb or scatter excitation light, leading to fluorescence quenching or background interference that lowers apparent recovery. Similarly, proteins and lipids in biological fluids such as serum can nonspecifically adsorb onto CDCQD surfaces, altering surface charge and blocking recognition sites, which reduces selectivity and signal reproducibility. These matrix effects highlight the importance of employing pretreatment strategies (e.g., filtration, dilution, solid-phase extraction) and surface engineering approaches (e.g., aptamer functionalization, antifouling coatings) to maintain reliable performance. Addressing these interferences is essential for translating CDCQD-based sensors from controlled laboratory environments to real environmental and biomedical applications.

### Environmental monitoring

Due to their environmentally friendly synthesis, high surface reactivity, and tunable fluorescence properties, carbohydrates-derived carbon quantum dots (CDCQDs) have become outstanding candidates for environmental monitoring. Their synthesis from renewable biomass not only supports sustainable development goals but also confers unique functionalities that allow for the sensitivity and selectivity of detecting a wide range of environmental pollutants. Recent developments in the use of CDCQDs for monitoring organic pollutants, volatile organic compounds (VOCs), hazardous gases, and water contaminants are highlighted in this section, with a focus on their analytical performance and practical applicability (Table 5).

**Table 5:** Comparative analysis of organic pollutants and gaseous pollutants detection using carbohydrate-based CQDs

Name and source	Analyte and detection limit	Ref
Laurel tree leaves (cellulose biomass)	Formaldehyde gas	Ayad, et al. (2023)
Holy basil (Tulsi) leaves (polysaccharide-rich biomass)	Ammonia (NH_3_ gas)	Alam et al. (2024)
Waste fruit peels (orange peel, watermelon peel)	Oxytetracycline (OTC), ~ 0.973 µM (orange-peel CQDs) and 0.077 µM (watermelon-peel CQDs); 0.25–100 µM (watermelon)	Gao et al. (2020)
Chitosan (biopolymer)	Nitrite (NO_2_⁻), LOD = 0.10 µM (100 nM)	Sun et al. (2021b)
Waste tobacco stems (cellulosic biomass)	Tetracyclines (TC, CTC, OTC), LOD = 1.328 nM (TC), 3.234 nM (CTC), 9.881 nM (OTC)	Yang et al. (2022)
Chitosan + 4-hydroxycoumarin (nitrogen sources)	Tetracycline (TC), Oxytetracycline (OTC), Chlortetracycline (CTC). LOD ≈0.12 mM (120–127 µM) for TC, OTC, CTC	Yang et al. (2022)
Glucose (hydrothermal synthesis)	Sudan I, Sudan IV (azo dyes) and Tetracycline HCl (antibiotic). LODs: 26.3 nM (Sudan I), 54.2 nM (Sudan IV), 31.1 nM (TC)	Zhang et al. (2022)
Sugarcane bagasse (cellulose waste, N-doped)	Tetracycline (antibiotic). LOD (linear range): 0–110 µM	Alfi et al. (2022)
CDs (CDs) with starch	Nitrobenzene (water), 14 μM	Wang (2021)
Chitosan-derived N-doped carbon dot	Nitrite (NO_2_⁻) (tap/lake water), LOD of 0.10 μM	Sun et al. (2021a)
Fish scale-derived carbon quantum dots (FS-CQDs)	The sensor exhibits a linear detection range of 0–150 μM and a LOD of 4.4 μM	Du et al. (2023)
Medicago sativa L (alfalfa)	Antibacterial drug nifuroxazide, 0.16 μM	Osman et al. (2024)
Ginkgo kernels	NO_2_^−^ in corn sausage, ham sausage, 0.15 μmol/L	Zhang et al. (2022)
Cellulose	NO_2_^−^, 1.060 μM	Hu et al. (2025)
Ziziphus Mauritiana	NH_3_, 10 nM	Ganesan et al. (2022b)
Orange peels	NO, 15 nM	Singh et al. (2023a)
Alkali lignin	Formaldehyde, 4.64 µM	Wang et al. (2021a)
Glucose	NO_2_^−^, 1.01 μM	Dong et al. (2025)
Xylan	NO_2_^−^, 0.36 μM	Chen et al. (2024)
Palm mane	Hg^2+^, 14.3 nM	Wu et al. (2022a)
Xylan	NO_2_^−^, 0.078 μM	Feng et al. (2022)

#### Air quality monitoring

CDCQDs have demonstrated substantial utility in air quality assessment, particularly for the detection of VOCs and toxic gases. For instance, laurel leaf-derived CQDs, when integrated into a quartz crystal microbalance (QCM) platform, enabled dual-mode gravimetric and fluorescence-based detection of formaldehyde-a prevalent and hazardous indoor VOC. This hybrid sensor exhibited linear frequency shifts and fluorescence responses proportional to formaldehyde concentration, maintaining selectivity even in humid environments and in the presence of interfering VOCs. Such performance underscores the potential of CDCQDs for real-time, reliable indoor air quality surveillance (Ayad, et al. 2023).

**Fig. 5:**
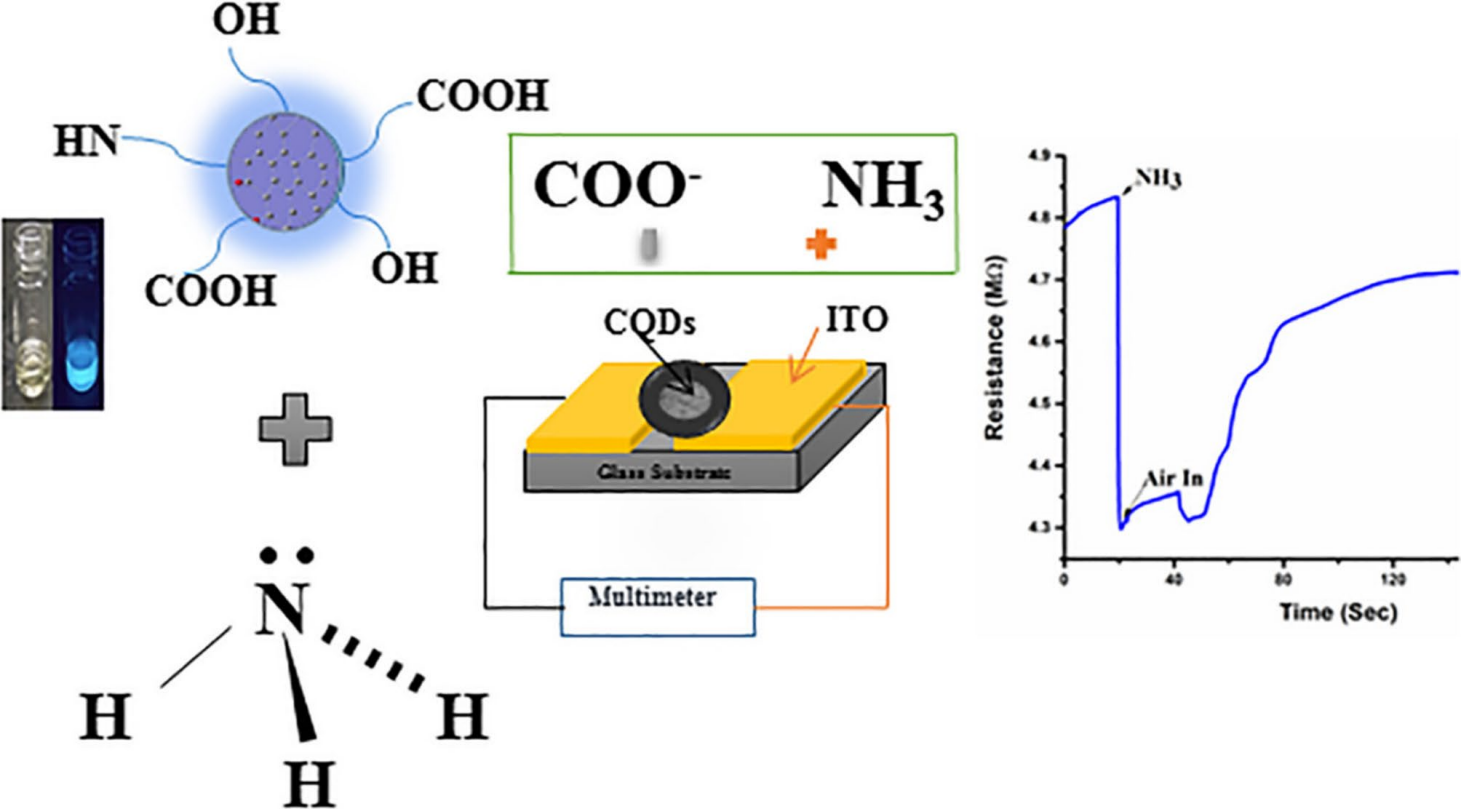
CQDs synthesized from holy basil (Tulsi) leaves in NH_3_ detection (Alam et al. 2024)

Similarly, CQDs synthesized from holy basil (Tulsi) leaves were tailored for ammonia (NH_3_) detection. The sensing mechanism relied on fluorescence quenching via specific interactions between surface functional groups and NH_3_ molecules. The sensor demonstrated rapid, reversible, and highly selective responses at low ppm concentrations, outperforming many conventional materials and highlighting its suitability for air safety applications (Fig. 5) (Alam et al. 2024).

#### Water quality assessment

Fruit peel-derived CQDs, like those from orange and watermelon, have been used for the fluorescence-based quantification of oxytetracycline (OTC) in tap water, lake water, and soil. These sensors achieved detection limits as low as 0.077 µM (watermelon-peel CQDs), with recovery rates between 91.7 and 103.2%, demonstrating both high sensitivity and practical feasibility. The use of CDCQDs in water quality monitoring has grown significantly and has shown remarkable success in detecting antibiotics, dyes, and inorganic ions in complex matrices (Gao et al. 2020).

Additionally, using the inner-filter effect, tobacco stem-derived CQDs allowed for the detection of several tetracycline antibiotics, including TC, CTC, and OTC, with recovery rates of up to 106% in water samples and nanomolar detection limits and wide linear ranges (Yang et al. 2022). Further detection of tetracycline and related antibiotics was demonstrated using chitosan + 4-hydroxycoumarin CQDs, which showed a quenching response modulated by Al^3^⁺ ions. However, their sensitivity was lower, with LODs in the 0.12 mM range, and linear ranges/recovery data were not fully specified (Yang et al. 2022). Using pH-dependent fluorescence quenching for sensitive and selective analysis, glucose-derived CQDs have also been used to detect food dyes (Sudan I and IV) and tetracycline hydrochloride in food matrices (Zhang et al. 2022).

Chitosan-based CQDs have been developed for the detection of nitrite (NO₂⁻) as one of inorganic pollutants, with a linear range of 1–500 µM and a detection limit of 0.10 µM. These sensors further demonstrated the resilience of CDCQD-based platforms by maintaining selectivity and accuracy in both tap and lake water (Zhang et al. 2022; Sun et al. 2021a).

#### Portable and low-cost sensing formats

The versatility of CDCQDs extends to portable and inexpensive analytical formats. For instance, nitrogen-doped CQDs made from sugarcane bagasse were used in paper-strip assays for tetracycline detection, providing a broad linear range (up to 110 µM) and strong selectivity, allowing for on-site, field-deployable testing (Alfi et al. 2022).; CQDs derived from fish scale were similarly adapted for the detection of toxic aquaculture dye malachite green, achieving detection limits of 4.4 µM and high recovery rates in real water and fish samples(Du et al. 2023); and starch-derived CQDs were used for nitrobenzene detection, exhibiting strong fluorescence quenching and linear response characteristics (LOD: 14 µM), making them appropriate for monitoring industrial pollution (Wang 2021).

#### Analytical performance and outlooks

When taken as a whole, these developments highlight the exceptional versatility and analytical capabilities of CDCQDs in environmental monitoring. Their capacity to identify a wide range of contaminants—such as dyes, antibiotics, volatile organic compounds, and inorganic ions—in real-world matrices with quick, reversible, and extremely sensitive reactions places them at the forefront of next-generation environmental surveillance. The incorporation of CDCQDs into portable, reasonably priced platforms further expands their usefulness and opens the door for their widespread use in pollution and public health protection.

Standardizing sensor production, guaranteeing batch-to-batch consistency, and incorporating these materials into intelligent, networked monitoring systems are still difficult tasks, nevertheless. For the commercialization of CDCQD-based environmental sensors, resolving these problems via sophisticated surface engineering, scalable green synthesis, and device miniaturization will be essential.

### Food safety and quality assurance

Because of their strong fluorescence, excellent biocompatibility, ease of surface modification, and environmentally friendly synthesis routes, CDCQDs have become very promising nanomaterials for food safety and quality, and they are also ideal candidates for integration into smart packaging systems and real-time monitoring of microbial contaminants, toxins, antibiotics, and spoilage indicators (Table 6).

**Table 6:** Comparative analysis of food contaminants detection using carbohydrate-based CQDs

Name and source	Analyte and detection limit	Reference
CDs (CDs) from carp roe	Tetracyclines (TC, CTC, OTC): 41.7 nM	Tang et al. (2022)
CDs (CDs) from Curcuma amada (mango ginger)	Tetracyclines: 33 nM (fluorescence), 0.5 nM (electrochemical) Fluoroquinolones: LOD = 2 nM	Korah et al. (2022)
Fe_3_O_4_@chitosan magnetic quantum dots	Salmonella typhimurium; Sample: lettuce (fresh-cut vegetable wash); LOD 138 CFU/mL in lettuce	Guo et al. (2020)
Orange peel (fruit biomass)	Escherichia coli O157:H7; Sample: milk; LOD 487 CFU/mL in milk	Hu, et al. (2021)
Sucrose derived CQDs	Escherichia coli O157:H7; Sample: milk; LOD ≈1 CFU/mL in milk	Yang, et al. (2019)
Carrot juice derived CQDs	Single-stranded DNA (ssDNA) probes to detect E. coli in food. LOD of 60 CFU/mL	Bai et al. (2023)
Hazelnut-husk CQDs	Aflatoxin B1 (AFB1) in corn, milk, peanuts; linear response ~ 25–250 ppm	Yuncu et al. (2024)
Crayfish-shell (chitin) CQDs	Thiamphenicol (amphenicol antibiotic) in animal-origin foods (e.g. milk, poultry). LOD ~ 11.12 µg/L; linear 20–300 µg/L	Chen, et al. (2414)
Green tea residue (food-processing waste)	Heavy metals (Hg^2+^, Pb^2+^, Fe^3+^, Cu^2+^) – Matrix: various real foods (five different foods tested). LOD on order of ~ 5 µM	Zhang et al. (2024d)
Paper-based CQDs	Five bacterial types (10^3^–10⁷ CFU/mL) via smartphone-based machine learning analysis	Zhu et al. (2025)
Moringa oleifera roots-based CQDs	Herbicide sulcotrione (LOD = 2 μg/mL)	Wang et al. (2021b)
Orange peel based CQDs	Escherichia coli O157:H7. 487 CFU/mL	Hu et al. (2021)
Cinnamomum tamala leaf extract CQDs	6.06 µM for ciprofloxacin (CPX)	Chaudhary et al. (2022)
Green CQDs	β-lactam antibiotic (amoxicillin) 1.01 × 10⁻^4^ M	Jessy Mercy et al. (2023)
Garlic skins CQDs	Fe^3^⁺; 4.44 μM	Zhai et al. (2022)
Lignin-derived CQDs	Anti-counterfeiting printing	Zhu et al. (2021b)
Vitex negundo leaves CDs	Food dye sunset yellow (LOD: 82.05 nM); Mn(II) with an LOD of 1.25 nM	Korah and Mathew (2023)
Coffee grounds CQDs	Sodium cyclamate, 3.16 µmol/L	Chen et al. (2021b)

#### Microbial pathogen detection

CDCQDs have been widely used in fluorescence-based sensing platforms for the sensitive and selective detection of foodborne pathogens; they are frequently combined with magnetic materials and aptamer or DNA probes to improve specificity and facilitate separation. For instance, a magnetic-fluorescent sensor made of Fe₃O₄@chitosan and aptamer-functionalized CQDs was able to detect Salmonella typhimurium in fresh-cut lettuce with a LOD of 138 CFU/mL through fluorescence quenching and magnetic separation (Guo et al. 2020). Similarly, CQDs derived from orange peels combined with complementary DNA-coated magnetic nanoparticles allowed for the detection of Escherichia coli O157:H7 in milk, with a LOD of 487 CFU/mL over a dynamic range of 5 × 10^2^ to 10^6^ CFU/mL (Hu, et al. 2021).

In addition, carrot juice-derived CQDs combined with single-stranded DNA probes allowed fluorescence recovery-based detection of E. coli with a LOD of 60 CFU/mL and a wide linear detection range from 10^2^ to 10⁸ CFU/mL (Bai et al. 2023). Interestingly, pH-sensitive ratiometric fluorescent sensors based on sucrose-derived CQDs showed exceptional sensitivity by exploiting metabolic acidification by E. coli O157:H7 to achieve an ultralow LOD of roughly 1 CFU/mL in milk (Yang, et al. 2019).

#### Detection of mycotoxins and antibiotics

CDCQDs have also been used to detect mycotoxins and antibiotic residues in food matrices with high sensitivity and accuracy. In antibiotic monitoring, chitin-based CQDs derived from crayfish shells achieved a LOD of 11.12 µg/L for thiamphenicol in milk and meat, with recovery rates ranging from 97.3 to 99.3% via static fluorescence quenching mediated by drug–CQD interactions (Chen, et al. 2414). Hazelnut husk-derived CQDs used Förster resonance energy transfer (FRET)-based quenching mechanisms to detect aflatoxin B1 (AFB1) in corn, milk, and peanuts, showing a linear detection range of ~ 25–250 ppm and recoveries between 81.6 and 98.6% (Yuncu et al. 2024).

CDCQDs are very sensitive and specific in antibiotic detection, which meets clinical diagnosis and drug quality control's imminent demands. For example, N,S-co-doped biomass CDs have shown superb selectivity toward tetracycline antibiotics (TC, CTC, OTC) based on an inner filter effect (IFE) with fast fluorescence response and excitation-independent emission at ~ 520 nm. CDs developed with carp roe obtained an LOD of 41.7 nM for tetracyclines, a wide linear range (0.1–50 µM), and high accuracy (98.6–104.5% recovery) in complicated matrices like serum, milk, and river water (Tang et al. 2022). Also, Curcuma amada-derived CDs provided dual-mode detection with LODs of 33 nM (fluorescence) and 0.5 nM (electrochemical) for tetracyclines and down to 2 nM for fluoroquinolones, constituting an exceedingly selective and sensitive platform for the analysis of antibiotics (Korah et al. 2022).

#### Multi-analyte sensing

CDCQDs' versatility includes simultaneous multi-analyte detection. Green tea residue-derived CQDs showed multiplexed sensing of heavy metals like Hg^2^⁺, Pb^2^⁺, Fe^3^⁺, and Cu^2^⁺ across a variety of food samples. Using chemometric algorithms (PSO-VWLS-SVM) to model fluorescence responses, this system achieved recoveries between 99.1 and 101.3% with LODs in the low micromolar range (Zhang et al. 2024d). To further enhance portability, a paper-based fluorescence sensor array containing Ag-, Cu-, and Zn-doped CQDs allowed for the quick identification of five bacterial species (10^3^–10⁷ CFU/mL) using smartphone-assisted machine learning. Within 30 min, the CQDs also demonstrated strong antibacterial activity, demonstrating the dual diagnostic and antimicrobial capabilities (Zhu et al. 2025).

For bacterial and antibiotic monitoring, CQDs derived from orange peel (Hu et al. 2021), Cinnamomum tamala leaves (Chaudhary et al. 2022), garlic skins (Zhai et al. 2022), and eco-friendly acid-doped lignin (Zhu et al. 2021b) showed strong fluorescence stability, good selectivity, and moderate to high QY (up to 43.4%). These materials were validated for the detection of ciprofloxacin (LOD = 6.06 µM), amoxicillin (LOD ≈ 0.1 mM), and Fe^3^⁺ (LOD = 4.44 µM), among others.

#### Dual-function sensors

In the context of food packaging, CDCQDs have been integrated into films and paper substrates to enable visual spoilage and contamination indicators. Garlic skin-derived CQDs embedded in poly(vinyl alcohol) films provided UV-blocking properties alongside strong fluorescence, making them suitable for real-time spoilage detection (Zhai et al. 2022).. Similarly, lignin-based CQDs exhibited tunable blue-to-green emission and pH-responsive photostability, facilitating applications in anti-counterfeiting inks and intelligent packaging. These multifunctional CDCQDs combine sensing and bioactivity (Zhu et al. 2021b). As another example, nitrogen-doped CQDs derived from Moringa root detected the herbicide sulcotrione at a LOD of 2 µg/mL while inhibiting fungal pathogens by over 75%, all without phytotoxic effects (Wang et al. 2021b). Last but not least, CQDs derived from Vitex negundo leaves were utilized to detect sunset yellow (LOD = 82.05 nM), Mn(II) (LOD = 1.25 nM), and worked as pH sensors and anti-counterfeiting inks, demonstrating the variety of food safety and quality applications that CQDs can support (Korah and Mathew 2023). Similarly, CQDs derived from coffee grounds allowed for the "turn-on" fluorescence detection of sodium cyclamate (LOD = 3.16 µM), well below the standard limits, emphasizing their use in sweetener monitoring (Chen et al. 2021b).

*Summary of *Sect. "Analytical chemistry applications"*:* Carbohydrate-derived carbon quantum dots (CDCQDs) have demonstrated broad and versatile applicability in analytical chemistry due to their tunable fluorescence, surface functionalization, and eco-friendly synthesis. In chemical sensing, CDCQDs enable ultrasensitive detection of heavy metals, pesticides, herbicides, and dyes, often reaching nanomolar or even picomolar limits of detection through mechanisms such as quenching, Förster resonance energy transfer (FRET), and turn-on/turn-off signaling. Their adaptability is further enhanced when integrated into hydrogels, composites, and portable formats, supporting environmental and field monitoring. In biomedical diagnostics, CDCQDs show high recovery rates and excellent biocompatibility for the detection of antibiotics, glucose, amino acids, vitamins, and other biomarkers, with potential integration into point-of-care devices. Similarly, in environmental monitoring, CDCQDs are effective in tracking pollutants such as antibiotics, volatile organic compounds, nitrites, and dyes, combining sensitivity with portability through paper strips and smartphone-readable outputs. In food safety, CDCQDs detect microbial pathogens, mycotoxins, antibiotics, and food dyes at extremely low concentrations, with promising use in smart packaging and dual-function antimicrobial systems. Collectively, these applications illustrate CDCQDs’ unique synergy of sensitivity, selectivity, and sustainability, positioning them as a next-generation platform for real-world diagnostic and monitoring solutions.

## Biocompatibility, safety evaluation, current challenges and future directions

### Biocompatibility and safety evaluation

CDCQDs must be biocompatible in order to be used in biomedical diagnostics, food safety monitoring, and environmental analysis. In vitro tests using human and animal cell lines (e.g., HEK293, SH-SY5Y, and MCF7) have generally shown low or negligible cytotoxicity at concentrations up to 200–400 µg/mL, depending on the source and surface chemistry of the CQDs. In vivo tests in zebrafish, mice, and nematodes have demonstrated good systemic tolerance, with minimal tissue accumulation or organ damage at low dosages. However, these evaluations heavily rely on physicochemical parameters like particle size, surface charge, and surface functional groups (Raju et al. 2024; Šafranko et al. 2021; Singh et al. 2023b).

When compared to other nanomaterials, this biocompatibility becomes more evident. Graphene quantum dots (GQDs) typically exhibit cytotoxicity at lower concentrations (~ 100–200 µg/mL), often due to sharper edges and higher oxidative stress potential. In contrast, metal nanoparticles (NPs) such as Ag or Au NPs demonstrate toxicity at even lower levels (20–50 µg/mL), primarily driven by ion release and reactive oxygen species (ROS) generation. The lower toxicity profile of CDCQDs can be attributed to their renewable carbohydrate origin, abundant surface –OH/–COOH groups, and the possibility of biocompatible passivation (e.g., PEGylation or amino acid capping). Thus, CDCQDs present a comparatively safer alternative to traditional nanomaterials, reinforcing their suitability for applications involving direct biological and environmental contact.

The incorporation of biocompatible passivating agents like polyethylene glycol (PEG), chitosan, or amino acids has been demonstrated to mitigate cytotoxic effects and enhance dispersion in biological fluids. Surface modifications greatly influence biocompatibility outcomes. Neutral or slightly negative zeta potentials, often achieved via carboxyl or hydroxyl functional groups, reduce interactions with negatively charged cell membranes and prevent hemolysis. Highly cationic CQDs may disrupt membrane integrity and increase toxicity. Smaller CQDs (< 10 nm) tend to exhibit better cellular uptake but may also present higher toxicity at elevated doses due to their ability to cross biological barriers (Singh et al. 2023c; Singh et al. 2025). These changes enable customized sensing and imaging applications in addition to enhancing cellular compatibility. As CQDs advance toward analytical application in the real world, regulatory considerations become more significant. CDCQDs must meet ISO/IEC and Good Laboratory Practice (GLP) requirements for analytical validation, including matrix compatibility, reproducibility, and stability, before being used in clinical diagnostics or food testing. In order to comply with the safety standards established by regulatory organizations like the U.S. FDA, EFSA, and OECD guidelines, thorough toxicological profile that takes into account genotoxicity, long-term exposure effects, and biodegradability is also necessary. Even though preliminary findings are encouraging, international regulatory frameworks and defined standards are still required to guarantee the consistent and safe deployment of CQDs in delicate human-related applications.

### Current challenges and future directions

Despite the unprecedented achievement and bright future of CDCQDs in analytical chemistry, several inherent difficulties must be addressed to make their widespread use and industrial upscaling possible.

While CDCQDs give excellent sensitivity and selectivity under tightly controlled lab conditions, their actions in complex real-world matrices such as biological fluids, foods, and environmental samples are of concern to analysts (Sohal et al. 2023; Tao et al. 2022; Wang et al. 2023b). Matrix interferences such as interference by concomitant ions, biomolecules, pH shift, and ionic strength drift can quench fluorescence signals and destroy detection accuracy. To surpass these limitations, follow-up work needs to focus on the rational design of CDCQDs with functionalized surface groups that target desired function and enhance selective binding while limiting nonspecific interaction. Additional implementation of multiplexed sensing platforms and novel signal amplification methods-such as ratiometric fluorescence probes and Förster resonance energy transfer (FRET)-based systems-will significantly augment sensitivity and robustness in multiplexed heterogeneous sample matrices.

The combination of CDCQDs with sophisticated analytical tools opens a revolutionizing pathway to compact, portable, and user-friendly diagnosis systems. Microfluidic chips, lab-on-a-chip technology, and smartphone-supported platforms are promising avenues of sensing miniaturization through wireless transmission of data and cloud computing-assisted analytics (Wareing et al. 2021; Wu et al. 2023; Wu et al. 2022b). Hybrid systems exploit the native photoluminescence and biocompatibility of CDCQDs and enhance the convenience of operation with decentralization of testing, especially beneficial under resource-limited or field conditions. Future work must focus on seamless integration protocols, device miniaturization, and creating robust software interfaces to effectively tap into these synergies.

Besides scientific and technological hurdles, commercialization of CDCQD-based sensors also demands overcoming regulatory, economic, and societal factors. Standardized characterization methods and rigorous eco-toxicology investigations need to be undertaken in order to establish safety profiles and gain regulatory approval. Long-term stability testing under different storage and operating conditions will also contribute to product reliability. Industry-academia interface can also promote technology transfer and facilitate incorporation of CDCQDs into existing diagnostic platforms and packaging systems. Optimization of cost structure and market strategy through economic studies will also be crucial for commercial success.

Finally, solving these sophisticated problems with interdisciplinary research and innovation will play a key role in realizing the complete potential of CDCQDs. With continuous development of large-scale synthesis, enhanced analytical performance, integration into technologies, and regulatory acceptance, CDCQDs are expected to revolutionize the field of environmentally friendly, high-performance nanobiosensors for ecological, biomedical, and food safety applications.

*Limitations and Practical Constraints:* Despite their outstanding analytical performance, CDCQDs face notable limitations that must be addressed before large-scale adoption. Scalability remains a challenge, as most hydrothermal and microwave-assisted syntheses are batch-based, limiting throughput compared to industrial nanomaterial production lines. Cost-effectiveness also raises concern: while precursors are inexpensive and renewable, the energy demand, specialized equipment, and need for surface functionalization can offset these advantages relative to traditional probes such as organic dyes or metal nanoparticles. Furthermore, batch-to-batch variability and lack of standardized protocols hinder reproducibility and regulatory approval. Acknowledging these limitations underscores that, although CDCQDs are promising, future work must focus equally on economic viability and industrial translation as on laboratory-scale sensitivity gains.

A major barrier to translating CDCQDs into real-world applications lies in regulatory approval processes. Agencies such as the FDA and EFSA require extensive toxicological, biodegradability, and long-term exposure data, which are still limited for CDCQDs. Standardized protocols for safety assessment and reproducibility are also lacking, delaying approval. On the industrial side, adoption rates remain slow due to uncertainties in large-scale synthesis, cost models, and integration into existing sensor technologies. Without clear regulatory frameworks and scalable production pipelines, the transition from laboratory research to commercial products will continue to face obstacles.

## Conclusions

CDCQDs have established themselves as one of the most challenging classes of nanomaterials in analytical chemistry on the strength of their renewable nature, optically tunable character, and exceptional biocompatibility. Between 2021 and 2025, massive research has established the unprecedented diversity of CDCQDs synthesized from a large range of carbohydrate sources-like monosaccharides (glucose, fructose), disaccharides (sucrose, lactose), polysaccharides (starch, chitosan), and biomass waste such as fruit peels, carrot juice, and sugarcane bagasse. This broad precursor range not only calls attention to the green chemistry guidelines driving this field but also enables tunable physicochemical properties to be tailored for specific analytical purposes.

Specifically, CDCQDs have demonstrated ultrasensitive detection levels across a broad range of applications. For example, laurel-derived CQDs enabled formaldehyde gas detection at low ppm levels (Ayad, et al. 2023), while fluorescence quenching was employed to rapidly sense ammonia (Alam et al. 2024). Watermelon skin-derived CDCQDs quantified oxytetracycline in water solution with a detection limit as low as 0.077 µM (Gao et al. 2020). In food safety, CQD-based biosensors have also exhibited great sensitivity levels by detecting E. coli O157:H7 in milk at 1 CFU/mL and Salmonella typhimurium in lettuce at 138 CFU/mL (Guo et al. 2020; Yang, et al. 2019). In biomedical diagnosis, CDCQDs have been able to detect glucose at 7 nM, ascorbic acid at 0.093 µM, and tetracycline antibiotics at 33 nM, highlighting their utility in clinical and pharmaceutical analysis (Patra et al. 2024; Liu et al. 2023b; Korah et al. 2022).

In addition to sensitivity, CDCQDs also demonstrate exceptional analytical robustness with typically documented recovery rates of 95% to 105% in complex real-world matrices such as blood, serum, milk, soil, and foodstuffs. They exhibit maximum QY as high as 83%, depending on the precursor and technique, and are highly photostable with uniform particle size (2–10 nm). Such characteristics collectively render CDCQDs highly suitable for use in portable, point-of-care sensing platforms.

In spite of these remarkable gains, numerous challenges remain to be overcome before CDCQDs achieve full commercial utility. The key challenges are scalable and reproducible synthesis, reproducible surface functionalization, and integration into plug-and-play devices. Overcoming these challenges via the development of environmentally benign continuous synthesis protocols, advanced surface chemistry, and integration into smart sensing technology-such as smartphone interfaces and microfluidics-will be essential. Further, rigorous regulatory testing and standardization are required to ensure consistency and make widespread implementation feasible.

Overall, CDCQDs embody the best synergy of sustainability, sensitivity, and specificity, providing detection ranges from picomolar to low micromolar concentrations, rapid response rates, and high flexibility to all environmental, biomedical, and food safety applications. Their continuous development and fruitful commercialization have a tremendous potential in revolutionizing dramatically the future design of eco-friendly, high-performance nanobiosensors, which in turn will result in more effective, inexpensive, and sustainable analytical tools.

## Data Availability

All data generated or analysed during this study are included in this published article.

## Declarations

### Human and animal participants

Not used human and animal subjects.
